# The mediating role of cognitive and affective empathy in the relationship of mindfulness with engagement in nursing

**DOI:** 10.1186/s12889-019-8129-7

**Published:** 2020-01-07

**Authors:** María del Carmen Pérez-Fuentes, José Jesús Gázquez Linares, María del Mar Molero Jurado, María del Mar Simón Márquez, África Martos Martínez

**Affiliations:** 1 Department of Psychology, University of Almería, 04120 Almería, Spain; 2 Universidad Politécnica y Artística del Paraguay, Asunción, Paraguay; 3 Department of Psychology, Universidad Autónoma de Chile, 4780000 Santiago, Chile

## Abstract

**Background:**

The work of health professionals is characterized by a high demand for psychological and emotional resources and high levels of stress. Therefore, the promotion of commitment and job well-being through strategies such as increased mindfulness, is important among nursing workers. Although mindfulness has shown positive effects in the health field, few studies have explored the mechanisms and processes underlying these results. We investigated the mediating role of empathy (cognitive/affective) in the effect of mindfulness on the dimensions of engagement in nursing professionals.

**Methods:**

Sample was comprised of 1268 Spanish nurses between 22 and 62 years old, that completed the Utrecht Labor Engagement Scale and the adapted versions of Mindful Attention Awareness Scale and Basic Empathy Scale. The relationship between variables to be included in the regression analyses, bivariate correlations were carried out, and the descriptive statistics of these variables were also found. To estimate the mediation model was used, in this case for multiple mediation effects.

**Results:**

Mindfulness is found to affect the Vigor and Dedication factors of engagement through cognitive empathy. While for the Absorption factor, the affective component of empathy also exerts a mediating role, although weaker than cognitive empathy. Cognitive empathy, as an individual factor, was shown to have a mediating effect between mindfulness and the factors of engagement in healthcare workers.

**Conclusions:**

The level of mindfulness influences engagement of nursing professionals positively, and this result is mediated mainly by cognitive empathy. Both mindfulness and empathy are modifiable individual factors, so their intervention by designing and implementing specific programs, can increase the commitment and wellbeing of professionals generating benefits to workers and to their patients.

## Background

Recent social and demographic changes have led to an increase in demand and challenges to the Spanish healthcare system. To be able to cope with them successfully, healthcare professionals, especially in nursing, must have strong work engagement (hereinafter, engagement) and provide quality care service [1, 2].

### The importance of mindfulness in nursing work

The work of health personnel is characterized by high psychological and emotional demands and a high level of perceived stress [3]. So the promotion of self-care and wellbeing of these professionals through strategies such as practicing mindfulness, become fundamental in maintaining optimum patient care [4–6].

The term mindfulness has been defined by Warren and Ryan [7, 8] as the ability of the individual to be fully aware and receptive to the events and experiences of the present moment. This experience of “being present” is possible thanks to the ability to become aware of themselves, attending to what is conscious and accepting what happens without judging. Beyond mindfulness as a concept based on individual experience, the practical implications of mindfulness can enhance the holistic practice of nursing care and the well-being of these workers [9]. Equally, mindfulness in these professionals is an effective strategy for prevention and management of anxiety, stress and exhaustion [10–13]. At the same time it improves their capacity for regulation and emotional wellbeing, which also has repercussions on the maintenance of successful and satisfactory therapeutic relations with their patients [14]. Therefore, mindfulness is a relevant concept within the nursing discipline due to its practical application to nursing professionals, optimum development of therapeutic action and integral promotion of their health [9]. It has also been demonstrated to generate positive effects on the organization after its implantation as a daily practice in the workplace [15]. Mindfulness programs for healthcare workers have been shown to have positive psychological effects on reducing emotional exhaustion [16–18]. It has also been reported to increase the ability to act consciously and satisfactorily in one’s job, and reduce reaction to inner experiences and exhaustion of nursing personnel [19].

Mindfulness perhaps does not provide a solution to many of the difficulties that arise in nursing work. However, its practice promotes the development of healthy coping strategies, increases resilience and work commitment and reduces the adverse effects of the demands of the profession [17, 20].

Concerning the variables involved, Hölzel et al. [21] proposed four mechanisms by which mindfulness is effective, one of which is emotional regulation. Along this line, the review by Gu, Stauss, Bond, and Cavanagh [22] found the beneficial effects of mindfulness practices to include mediating variables such as psychological flexibility, emotional regulation, clarifying inner values and absence of attachment and reaction.

### Engagement in nursing professionals

Engagement refers a positive mental state and of job commitment characterized by the vigor, dedication and absorption dimensions [23], which generate strong personal initiative [24] and optimum development of the task [25]. Vigor provides high energy and professional resistance, dedication refers to the experience of challenge and enthusiasm in the work environment, and absorption is characterized by total concentration on one’s work [26].

Organizational factors significantly influence the engagement of nursing professionals [27]. However, some individual variables have also been associated with the development of participation in health workers. Among them, self-efficacy [28] and emotional intelligence [29, 30]. Participation in nursing improves the quality of service and care. Although contextual and dispositional factors intervene in this relationship [31].. It is also associated with greater job satisfaction and less desire to change in nursing personnel [32–34]. So engagement can affect the work performance of nursing workers and the results of patients and the organization [27].

Within the organizational sphere, training in mindfulness has been associated with increased engagement, job satisfaction and performance [35–37], results which have been repeated with nursing professionals [38, 39]. With health sciences students, mindfulness showed a strong association with engagement [40], such that intervention improved engagement in future healthcare professionals [41]. Similarly, the practice of mindfulness, especially when done on a regular basis, has been shown to have a significant impact on the stress, anxiety, exhaustion and well-being of nursing professionals. What affects their working life [14], reducing organizational costs and improving the quality and safety of patient care [5].

### Empathy in healthcare professionals and their relationship with mindfulness and engagement

Empathy is an individual variable recognized by nursing personnel themselves as indispensable for proper performance of their professional labor [1]. Beyond simple identification of the emotional state of another person, empathy involves experiencing this state based on its two dimensions, affective and cognitive [42]. Within the health context and at the factor level, the emotional component of the empathy refers to the professional’s ability to experience and share the patient’s feelings. While the cognitive component encompasses the identification and intellectual understanding of the feelings of the other, from a perspective objective [43]. However, authors such as Schwan [44] argue that the affective component of empathy may lead to responsibility and excessive worry about the patient more than benefit proper practice.

Furthermore, certain variables, such as empathy and experience are at the basis of interpersonal therapeutic relations and engagement in nursing practice [45]. Concerning the relationship between empathy and engagement, unlike the affective component, the cognitive component of empathy has been associated with increased job satisfaction, self-efficacy and engagement [46, 47]. In the same line, perspective as a factor of cognitive empathy has been related to absorption in healthcare workers. Thus, those professionals with a greater capacity to see from the patient’s point of view become involved more with their work, and are able to keep up stronger concentration. And, on the contrary, emotional empathy shows a negative association with the dedication factor of engagement [48]. The lack of empathy in this sector may generate high levels of emotional exhaustion [49]. Even though empathy is a main component of nursing practice, it is also the core of feelings of guilt, which when they are excessive or poorly directed, can lead to beliefs of responsibility which generate high distress in professionals [50]. There are therefore problems for establishing a balance between detachment and emotional connection with the patient in clinical practice [51], which could derive in a loss of wellbeing and quality of care, generating stress, exhaustion and avoidance coping styles [37]. In this line, some studies have suggested the suitability of training cognitive and behavioral empathy in nurses to improve the care given patients and the efficacy of their services. Whereas, on the contrary, the increase in emotional empathy, could imply identifying too closely the emotions of the patient and the family, increasing anxiety and exhaustion in health professionals [44, 52, 53]. Thus, healthcare professionals who understand their patients and families but maintain a certain emotional distance are able to cope with the emotional demands of their job successfully [54]. Empathy in healthcare personnel improves with increased mindfulness [55, 56]. So, being focused on the present moment, helping to calm the mind and emotions, improving the ability to care for distressed patients without suffering emotional contagion [57]. Therefore, mindfulness practice by workers in this sector facilitates awareness and being in the present moment, enabling recognition of the empathetic experience and promoting critical examination [58].

Improvement in the work climate and nursing action derive from both progress in working conditions and developing individual internal resources, maybe which, in turn, foster coping with highly stressful conditions, exhaustion and emotional impact [16, 59–61]. As mindfulness is a potentially modifiable individual characteristic, its intervention can generate positive results in the scope of healthcare [62]. However, as mentioned by Duarte and Pinto-Gouveia [63], even though mindfulness-based intervention has received much empirical support due to its efficacy, few studies have explored the underlying mechanisms and processes by which mindfulness generates these results. Therefore, the objective of this study was to analyze the mediating role of empathy (cognitive/affective) in the effect of mindfulness on the dimensions of engagement in nursing professionals.

**Fig. 1:**
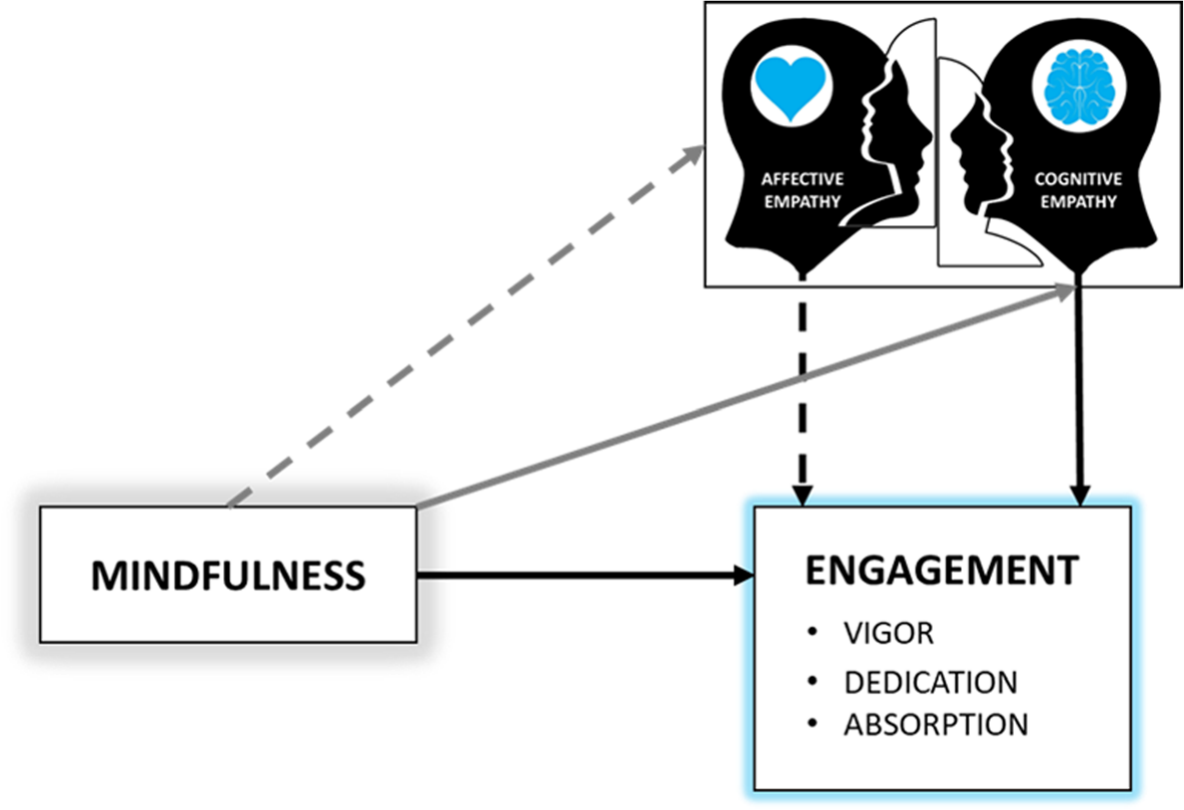
Hypothetized model of the mediation effects of empathy on the relationship between mindfulness and engagement

Thus, according to previous studies, levels of cognitive empathy influence the commitment of health workers [46, 47, 64]. On the other hand, although the positive effects of mindfulness on empathy have been evidenced in the literature [65], especially on the more cognitive aspects of this construct [56], and on engagement [17, 66], so far the effects of mediation between these variables have not been assessed. Therefore, a model is developed where the previously stated relationships are hypothesized (Fig. 1).

## Methods

The study employed a cross-sectional descriptive and correlational design. A questionnaires survey approach was conducted in 2017. With the general objective of analyzing the relationships between mindfulness and engagement components, attending to possible mediating effects of empathy. Specifically, in the group of nursing professionals, that carry out their work in a hospital environment.

### Participants

The initial sample was made up of 1383 nurses in Andalusia (Spain). As the main variable in the study was work engagement, data on professionals who were unemployed (68 subjects) at the time of study were discarded. Any questionnaires in which random or incongruent answers were found from the control questions (CQ) inserted for the purpose were also eliminated. There were five subjects with erroneous answers on CQ2, 32 who missed CQ3, and 10 subjects on CQ5. The final sample was therefore comprised of 1268 Spanish nurses aged 22 to 63, with a mean age of 32.02 years (*SD* = 6.91). 14.7% (*n* = 187) of the sample were men and 85.3% (*n* = 1081) were women with mean ages of 32.79 (*SD* = 6.27) and 32.24 (*SD* = 6.68), respectively. The sample and characteristics distribution are shown in Table 1.

**Table 1:** Distribution and sample characteristics (*N* = 1268)

	n	%
Sex		
Women	1081	85.3%
Men	187	14.7%
Age (years)		
≤ 30	645	50.9%
> 30	623	49.1%
Marital status		
Single	699	55.1%
Married	534	42.1%
Separated/Divorced	34	2.7%
Widowed	1	0.1%
Work area		
Floor	457	36%
Emergencies	284	22.4%
Intensive Care Unit	133	10.5%
Operating room	132	10.4%
Mental Health Unit	46	3.6%
External consults	44	3.5%
Other	172	13.6%
Work shift		
Rotating	943	74.4%
24 hours	41	3.2%
Night	37	2.9%
Morning or evening	247	19.5%

### Instruments

The Spanish version by Soler et al. [67] of the *Mindful Attention Awareness Scale* (MAAS) [7] was used to evaluate mindfulness. This scale, which assesses receptive awareness in the present moment consists of 15 items with Likert-type answer choices from 1 (always) to 5 (never). The score is obtained from the arithmetic mean of the total items and high scores indicate greater mindfulness status. Reliability found for this study was adequate (α = .89), coinciding with the alpha found by the authors of the scale’s validation [67]. This Spanish version of the scale has been used in the sample of nurses [68], obtaining the same value of alpha .89.

The *Utrecht Work Engagement Scale* (UWES) [69] was applied to assess engagement. This scale is a self-report instrument which measures work commitment. It consists of 17 items which the subject must answer on a six-point Likert-type scale (0–6). It provides information on three dimensions: Vigor, Dedication and Absorption. The scale’s psychometric properties were adequate for reliability and validity [69], and has been widely used in the nursing community [70]. In this study, internal reliability on each of the subscales was .89 for the Vigor dimension, .91 for Dedication and .85 for Absorption.

Finally, the adaptation by Oliva et al. [71] of the *Basic Empathy Scale* (BES) [72] was used to measure empathy. It is based on the definition of empathy proposed by Cohen and Strayer [73], that is, the act of understanding and sharing the emotional context of another person. It has nine items distributed in two sub-scales corresponding to affective and cognitive empathy, answered on a five-point Likert-type scale (1–5). Higher scores are interpreted as indicating more intense empathic behavior. The internal consistency reported by Oliva et al. [71], in adolescents, for both scales was .73 and .63 respectively. In this study, in a nurse’s sample, the internal consistency found with the Cronbach’s alpha was .84 on both scales.

### Procedure

Prior to collecting data, we assured the participants that the treatment of data in the study would comply with applicable standards of data security, confidentiality and ethics. The pertinent permissions were requested on an informed consent sheet addressed before the tests were implemented. The study was approved by the Bioethics Committee of the University of Almería (Spain). The application of the questionnaire was done through a web platform which allowed subjects to complete them online. A series of control questions were included to monitor for random or incongruent responses, which were removed from the study databased.

### Data analysis

First, to test the relationship between variables to be included in the regression analyses, bivariate correlations were carried out, and the descriptive statistics of these variables were also found. Normality tests are applied (Kolmorov-Smirnov). To verify the assumptions of collinearity in the data, regression analysis and estimation of the Tolerance coefficients and Inflation Factors of Variance (VIF) were carried out. The hypothesis of non-multicollinearity is not violated, obtaining condition indices that do not exceed 30 [74].

To estimate the mediation model, the SPSS macro by Hayes [75] was used, in this case for multiple mediation effects [76]. This resource enables computation of regression models to find information on indirect effects, avoiding the limitations of the classic proposal by Baron and Kenny [77]. Specifically, the distinction between mediation and indirect effect is not always made from the proposal of Baron and Kenny, therefore, the search for evidence of indirect effects can be terminated, if there is no evidence that X and Y are associated. As Hayes [78] suggests, if the size of the total effect limits the size of the indirect effects and, therefore, its product, this logic would make sense. In this sense, the claim that X cannot affect Y indirectly in the absence of a total detectable effect is false.

Bootstrapping (5000 bootstrap samples) applied for this enabled estimation at 95% confidence intervals and determination of the mediation effect. In this case, a multiple mediation analysis was performed with two mediators operating in series. It is a method intended for path analyses that are identifying two mediator variables along different paths.

The mediation analysis was carried out based on the following mediational hypothesis: The level of mindfulness positively influences engagement of nursing professionals, mediated by empathy, mainly cognitive.

To compute the model, the total score on the MAAS was taken as the measure of the level of mindfulness, and was entered as the independent or predictor variable. The three dimensions of engagement were proposed as the dependent variable for each of the models and finally, Cognitive Empathy (M1) and Affective Empathy (M2) as the mediator variables. A multiple mediation model with two mediators operating in series was thus computed (M1: C-E and M2: A-E).

## Results

### Descriptive and correlational analyses of engagement, empathy and mindfulness

Table 2 shows the descriptive statistics and correlations between the study variables: Engagement (Vigor, Dedication & Absorption), Empathy (Cognitive & Affective), and Mindfulness. The study sample obtains the following average scores in the engagement components (with a range of 0–6): Vigor (*M* = 4.46, *SD* = 1.06), Dedication (*M* = 4.45, *SD* = 1.17), and Absorption (*M* = 4.00, *SD* = 1.09). Regarding the dimensions of Empathy, an average of 19.36 (*SD* = 2.55, range 5–25) is obtained in Cognitive Empathy, and an average of 13.36 (*SD* = 3.02, range 4–20) in Affective Empathy. On the other hand, the average in mindfulness for the study sample is 3.64 (*SD* = .55), in a range of 1–5.

**Table 2:** Descriptive statistics and correlations: Engagement, Empathy and Mindfulness

	*M*	*SD*	1	2	3	4	5	6
1. Vigor	4.46	1.06	–					
2. Dedication	4.45	1.17	.90^***^	–				
3. Absorption	4.00	1.09	.83^***^	.82^***^	–			
4. Cognitive empathy	19.36	2.55	.33^***^	.28^***^	.25^***^	–		
5. Affective empathy	13.36	3.02	.07^**^	.04	.12^***^	.33^***^	–	
6. Mindfulness	3.64	.55	.30^***^	.32^***^	.13^***^	.12^***^	−.17^***^	–

Note: ^***^p < .001

First, it is observed that the level of Mindfulness correlated positively with the three dimensions of engagement (VI: *r* = .30, *p* < .001; DE: *r* = .32, *p* < .001; AB: *r* = .13, *p* < .001).

On the other hand, for the relationships between Mindfulness and the two types of Empathy, data confirmed the existence of a positive correlation with Cognitive Empathy (*r* = .12, *p* < .001), while it was negative with Affective Empathy (*r* = −.17, *p* < .001).

Finally, positive correlations were found between the dimensions of Engagement and Cognitive Empathy (VI: *r* = .33, *p* < .001; DE: *r* = .28, *p* < .001; AB: *r* = .25, *p* < .001). Affective Empathy was positively correlated with Vigor (*r* = .07, *p* < .01) and Absorption (*r* = .12, *p* < .001), but there was no correlation with Dedication (*r* = .04, *p* = .10).

### On the mediating effect of cognitive and affective empathy in the relationship between mindfulness and engagement: multiple mediation analysis

**Fig. 2:**
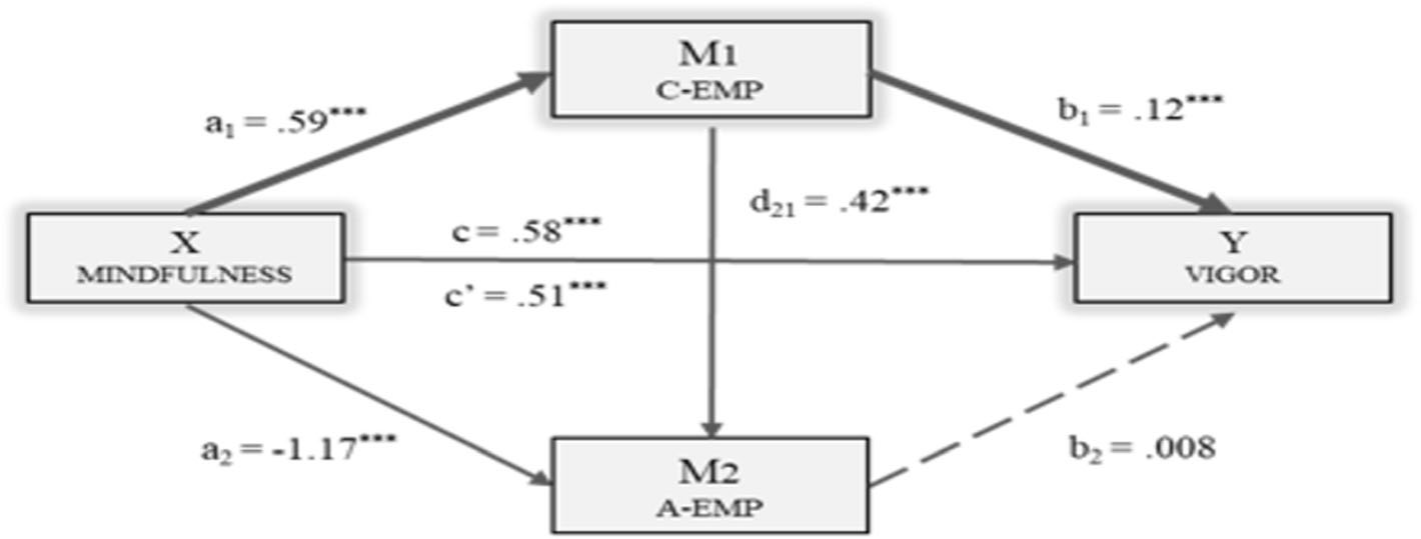
Multiple Mediation Model of Empathy (cognitive/affective) on the relationship between Mindfulness and Vigor. Note. C-EMP = Cognitive Empathy; A-EMP = Affective Empathy. ***p < .001

**Fig. 3:**
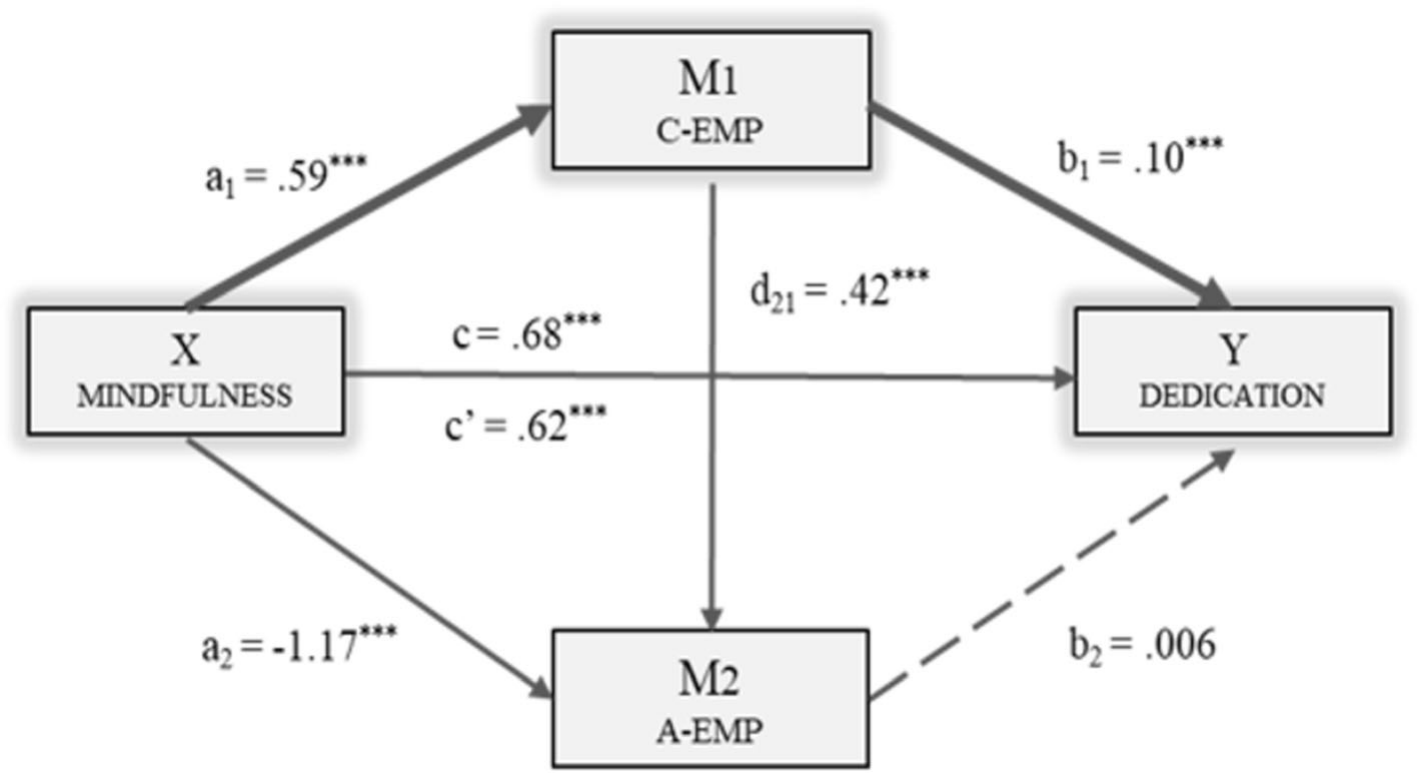
Multiple Mediation Model of Empathy (Cognitive/Affective) in the relationship between Mindfulness and Dedication. Note. C-EMP = Cognitive Empathy; A-EMP = Affective Empathy. ***p < .001

**Fig. 4:**
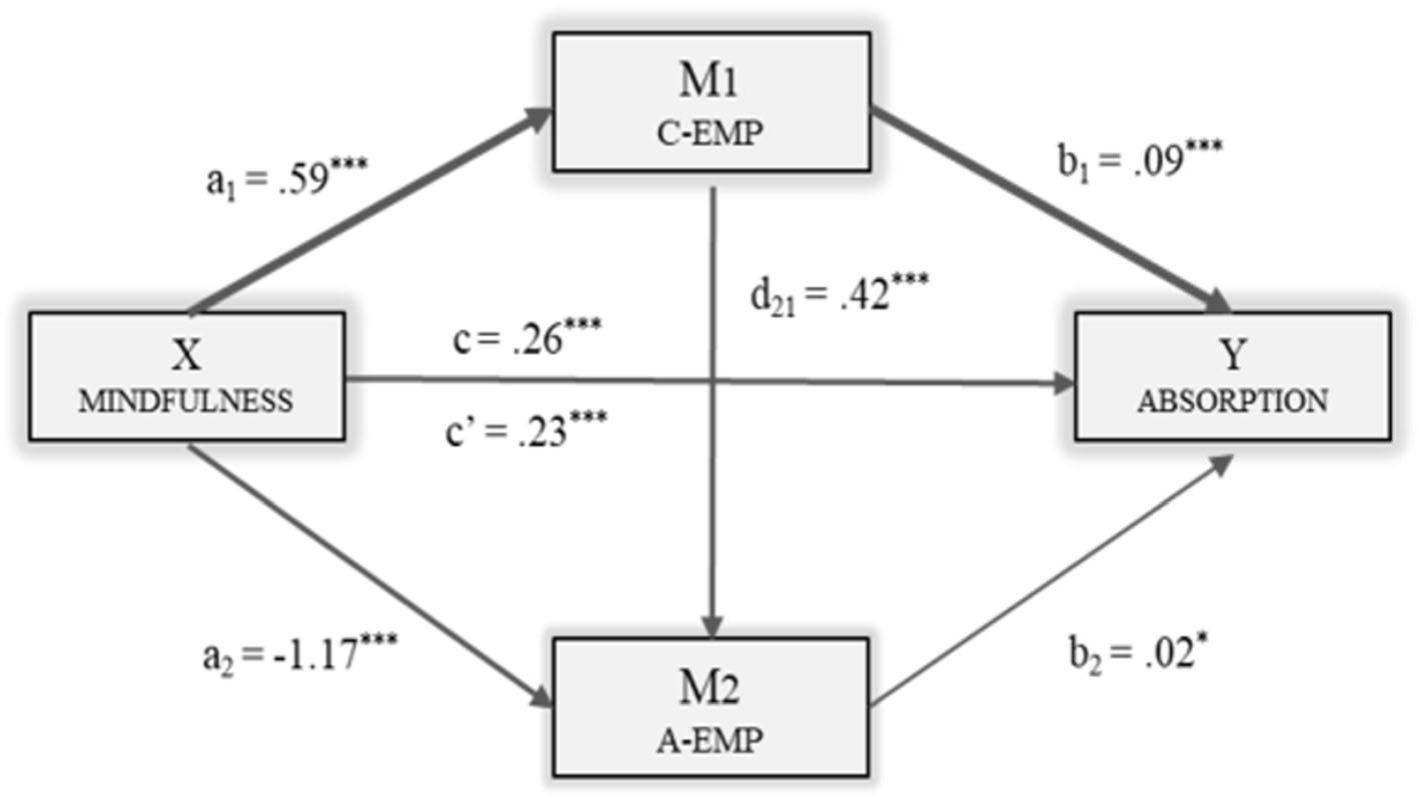
Multiple Mediation Model of Empathy (Cognitive/Affective) on the relationship between Mindfulness and Absorption. Note. C-EMP = Cognitive Empathy; A-EMP = Affective Empathy. ***p < .001, *p < .05

The following figures show the models in which each of the dimensions of engagement was included as the dependent variable: Vigor (Fig. 2), Dedication (Fig. 3) and Absorption (Fig. 4). The direct, indirect and total effects were analyzed for each case.

First of all, a statistically significant effect [BMindF = .59, *p* < .001] was observed for Mindfulness (X) on Cognitive Empathy (M1). The effect of Cognitive Empathy [BC-EMP = .42, *p* < .001] and Mindfulness [BMindF = − 1.17, *p* < .001] on Affective Empathy (M2) was also significant.

The third regression analysis took Vigor as the result variable (Y), estimating the effect of the independent variable and of the two mediator variables (Fig. 2). In this case, significant effects of Cognitive Empathy [BC-EMP = .12, *p* < .001] and Mindfulness as the independent variable [BMindF = .51, *p* < .001]. Affective Empathy (M2) did not show any significant effect [BA-EMP = .008, *p* = .366] on the dependent variable. The total effect of Mindfulness on Vigor was significant [BMindF = .58, *p* < .001].

An analysis of indirect effects was also carried out using bootstrapping, finding data in support of a significant level for Path 1 [Ind1: X → M1 → Y; B = .07, SE = .02, 95% CI (.03, .11)]. Therefore, Mindfulness had a stronger effect on Vigor through Cognitive Empathy (M1) than both mediators operating in series.

Figure 3 shows how taking Dedication as the result variable (Y), a significant effect was found for both Cognitive Empathy [*B*_C-EMP_ = .10, *p* < .001] and Mindfulness [*B*_MindF_ = .62, *p* < .001]. However, no significant effect was observed of Affective Empathy (M_2_) [*B*_A-EMP_ = .006, *p* = .531] on Dedication. The total effect of Mindfulness on Dedication was significant [*B*_MindF_ = .68, *p* < .001].

The data derived from analysis of indirect effects suggest statistical significance of Path 1 [Ind_1_: X → M_1_ → Y; *B* = .06, *SE* = .01, 95% *CI* (.03, .10)]. Therefore, the effect that Mindfulness had on Dedication was stronger through Cognitive Empathy (M1) than through involvement of the two mediators.

Finally, as shown in Fig. 4, including Absorption in the model as the dependent variable (Y), the data revealed a significant effect of the independent variable [*B*_MindF_ = .23, *p* < .001] and also of the two mediators separately [*B*_C-EMP_ = .09, *p* < .001; *B*_A-EMP_ = .02, *p* < .05] on the dependent variable. The total effect of Mindfulness on Absorption was significant [*B*_MindF_ = .26, *p* < .001].

Finally, the analysis of indirect effects using bootstrapping provided data suggesting that although the effect of Mindfulness was statistically significant by Paths 2 [Ind_2_: X → M_1_ → M_2_ → Y; *B* = .006, *SE* = .003, 95% *CI* (.001, .015)] and 3 [Ind_3_: X → M_2_ → Y; *B* = -.03, *SE* = .01, 95% *CI* (−.059, −.004)], it was Path 1 [Ind_1_: X → M_1_ → Y; *B* = .05, *SE* = .01, 95% *CI* (.02, .09)] which was the most significant. Therefore, both Cognitive and Affective Empathy exerted a mediating role in the Absorption dimension, but it was Cognitive Empathy (M1) which showed the main indirect effect in the model.

## Discussion

According to the results of this study, nursing professionals showed a positive relationship between Cognitive Empathy and Engagement and Mindfulness. While Affective Empathy had a positive association with the Vigor and Absorption factors of Engagement and negative with Mindfulness. Thus, critical, conscious recognition of feelings of empathy is favored by increased mindfulness [55–58]. This in turn is associated with a reduction of adverse effects of the professional demand, increasing work commitment [17, 20]. Likewise, mindfulness, generates a reduction in emotional reaction to internal experiences [19] so its association with Affective Empathy in healthcare professionals is, up to a certain point, negative. However, unlike other studies [48], the scores on the Dedication factor did not show any relationship with Affective Empathy.

This study also analyzed the mediating role of empathy in the effect of mindfulness on engagement of healthcare workers. Studies on the mechanisms of change underlying the effects of mindfulness on engagement of nursing professionals is a crucial task for improving the quality, execution and efficacy of intervention [63]. In this light, the level of mindfulness influences engagement of nursing professionals positively, and this result is mediated mainly by cognitive empathy. Thus, mindfulness is found to affect the Vigor and Dedication factors of engagement through cognitive empathy. While for the Absorption factor, the affective component of empathy also exerts a mediating role, although weaker than cognitive empathy. Accordingly, the capacity to regulate one’s own emotions and achieve absence of attachment and reaction to the affective state of the patient, would be mediating mechanisms in the achievement of positive results of mindfulness [21, 22].

Finally, in the same line of the study by Navarro-Abal, López-López, and Climent-Rodríguez [48], the results showed a mediating effect of the cognitive factor of empathy on Absorption. Since this factor refers to mindful attention while working [26], it was expected that this component of engagement, in which affective empathy exerts a mediating role, would require total attention to the task being performed, impede evading identification with the emotional state of the patient [43], and hinder the balance between detachment and emotional connection necessary for clinical practice [51].

Among the limitations of this study is the disparity between sexes in the sample. However, this is a characteristic typical of the population studied in which there are more women. Neither was the area where they were performing their duties taken into consideration, which could relate strongly to the feelings of empathy developed with the patient or their families. It would therefore be appropriate to consider this variable in future research.

Thus, as pointed out by different studies [46, 47], the cognitive factor of empathy affects the level of engagement of health workers. And, according to our results, I would be involved as a mediator in the relationship between mindfulness and work commitment. Meanwhile, the affective factor can generate a high level of concern and guilt among these workers [44], impeding work performance. Therefore, its inclusion as a mediator of the relationship between mindfulness and engagement would be limited to the absorption factor, and with a force much more limited than that of cognitive empathy.

In this way, healthcare professionals experience a wide range of emotional, psychological and physical demands, in addition to being safe at high levels of stress [3]. This may affect the quality of patient care, as well as the worker’s own well-being and commitment [1, 2]. Mindfulness-based considerations have demonstrated their positive effects on the work commitment of health personnel [17, 66]. And about critical rates for effective patient care such as empathy [56, 65]. However, so far, studies that have explored the influence of mindfulness have a limited impact, since they have not addressed the effects that mediate this relationship [63]. The present study represents an advance in the knowledge of this association, exploring the ways by which the variables of the study in nursing professionals are related. And therefore, it can contribute to the development of specific associations.

## Conclusions

Engagement in healthcare professionals is a highly desirable due to the many benefits it generates for professionals in all sectors, but especially, in healthcare workers, and mindfulness has been related to its development. However, even though the favorable effects of mindfulness on healthcare professionals are widely known, little is known about the underlying mechanisms. Cognitive empathy, as an individual factor, was shown to have a mediating effect between mindfulness and the factors of engagement in healthcare workers.

The results found concur in the need to continue inquiring into the factors which mediate the effects of mindfulness. Both mindfulness and empathy are modifiable individual factors, so their intervention by designing and implementing specific programs, at takes into account the mediation effects found, can increase the commitment and wellbeing of professionals generating benefits to workers and to their patients.
